# An international study on emerging arboviral infections and blood safety

**DOI:** 10.1111/trf.70306

**Published:** 2026-06-18

**Authors:** Piya Rajendra, Helen M. Faddy, Daniel Candotti, Carolina B. Bub, Jose M. Kutner, Steven J. Drews, Carmen L. Charlton, Sheila F. O'Brien, Wai‐Chiu Tsoi, Michel‐Andres Garcia‐Otalora, Claire Reynolds, Tuulia Palukka, Riikka Lehtisalo, Ruth Offergeld, Axel Seltsam, Marijke Weber‐Schehl, Nahid Chenarsabz, Naoko Goto, Sung‐Chan Park, So‐Yong Kwon, Ryanne Lieshout‐Krikke, Aneta Kopacz, Ewa Sulkowska, Oana‐Alexandra Șerban, Simona Ruta, Eugene Zhiburt, Maha Badawi, Josephine Mitchel, Ute Jentsch, Ángel Luis Pajares Herraiz, Ana Lafuente Guijosa, Gülşen Özkaya Şahin, Christoph Niederhauser, Marion C. Lanteri, Brian Custer, Galen Conti, Anthea Cheng, Shannah Secret, Peter Simmonds, Heli Harvala, the Virology subgroup of the ISBT WP‐TTID

**Affiliations:** ^1^ Radcliffe Department of Medicine University of Oxford Oxford UK; ^2^ School of Health University of the Sunshine Coast Petrie Queensland Australia; ^3^ Research and Development Australian Red Cross Lifeblood Brisbane Queensland Australia; ^4^ Institut Mondor de Recherche Biomédicale, INSERM U955, Team Virus‐Hepatology‐Cancer, Henri Mondor Hospital University Paris‐Est Paris France; ^5^ Hospital Israelita Albert Einstein São Paulo Brazil; ^6^ Medical Microbiology and Surveillance & Discovery Laboratory, Canadian Blood Services, Division of Diagnostic and Applied Microbiology University of Alberta Edmonton Alberta Canada; ^7^ Hong Kong Red Cross Blood Transfusion Service Hong Kong China; ^8^ School of Medicine and Health Science Universidad del Rosario Bogotá Colombia; ^9^ NHS Blood and Transplant Colindale UK; ^10^ Finnish Red Cross Blood Service Helsinki Finland; ^11^ Department of Infectious Disease Epidemiology Robert Koch Institute Berlin Germany; ^12^ Bavarian Red Cross Blood Service Institute Wiesentheid Wiesentheid Germany; ^13^ Iranian Blood Transfusion Organization Iran; ^14^ Japanese Red Cross Society Blood Service Japan; ^15^ Korean Red Cross Blood Services Wonju Korea; ^16^ Department of Medical Affairs Sanquin Blood Supply Foundation Amsterdam The Netherlands; ^17^ Institute of Hematology and Transfusion Medicine Warsaw Poland; ^18^ National Institute “Prof. Dr. C.T. Nicolau” Bucharest Romania; ^19^ School of Advanced Studies of the Romanian Academy Bucharest Romania; ^20^ Stefan S. Nicolau Institute of Virology Bucharest Romania; ^21^ “Carol Davila” University of Medicine and Pharmacy Bucharest Romania; ^22^ Pigorov National Medical Surgical Center Russia; ^23^ Department of Hematology, Faculty of Medicine King Abdulaziz University Jeddah Saudi Arabia; ^24^ South African Blood Transfusion Services South Africa; ^25^ Centro de Transfusión de la Comunidad de Madrid Madrid Spain; ^26^ Clinical Microbiology, Infection Prevention and Control Office for Medical Services Lund Sweden; ^27^ Division of Medical Microbiology, Department of Laboratory Medicine Medical Faculty, Lund University Malmö Sweden; ^28^ Interregional Blood Transfusion SRC Bern Switzerland; ^29^ Department of Laboratory Medicine University of California San Francisco San Francisco California USA; ^30^ Creative Testing Solutions Tempe Arizona USA; ^31^ Vitalant Research Institute San Francisco California USA; ^32^ American Red Cross Biomedical Services Medical and Scientific Office Washington, DC USA; ^33^ Donor and Product Safety Policy Unit Australian Red Cross Lifeblood Perth Western Australia Australia; ^34^ Nuffield Department of Medicine University of Oxford Oxford UK; ^35^ Institute of Biomedicine, Medical Faculty University of Turku and Turku University Hospital Turku Finland; ^36^ Microbiology Services NHS Blood and Transplant London UK

## Abstract

**Background:**

Emerging and re‐emerging arboviral infections are a risk to blood safety. We conducted an international survey on how blood establishments respond to current and future arbovirus threats.

**Study Design and Methods:**

A questionnaire on arbovirus donor deferral strategies, pathogen reduction, and donation screening was distributed to members of the International Society of Blood Transfusion working party on transfusion‐transmitted infectious diseases. Data from 2024 were gathered and analyzed.

**Results:**

A total of 23 survey responses were received from 21 countries. This covered a population of 1.45 billion people and 29.9 million blood donations collected in 2024. All respondents applied travel‐based donor deferrals, whereas pathogen reduction, implemented by half of the respondents, was mostly applied for a selection of plasma and platelet donations. West Nile virus (WNV) was the only arbovirus blood donations were screened for by nine respondents from eight countries, with 256 donations confirmed as WNV RNA‐positive in 2024. No transfusion‐transmitted WNV infections were reported.

**Discussion:**

Blood safety measures remain limited and unevenly distributed globally, and in their present form, are unlikely to provide protection against the growing range of emerging arboviruses. Donor deferral may not always be a sustainable blood safety strategy alone for all blood operators, due to large‐scale outbreaks associated with these viruses. While pathogen reduction methodologies are being developed to be applied to all blood components, risk assessments for (re)‐emerging arboviruses, such as dengue, chikungunya, and Zika viruses, should be performed to determine if additional mitigation, such as blood donation screening, is warranted.

**Abbreviations**
CHIKV: chikungunya virus
DENV: dengue
FDA: Food and Drug Administration
ID‐NAT: individual nucleic acid amplification testing
ISBT: International Society of Blood Transfusion
JEV: Japanese encephalitis virus
NAT: nucleic acid amplification testing
NGS: next generation sequencing
NK: not known
PDMPs: plasma‐derived medicinal products
RBC: red blood cells
RNA: ribonucleic acid
TBEV: tick‐borne encephalitis virus
UK: United Kingdom
USA: United States of America
USUV: Usutu Virus
WHO: World Health Organization
WNV: West Nile Virus
WP‐TTID: working party on transfusion‐transmitted infectious disease
ZIKV: Zika

## 1 INTRODUCTION

The global (re)‐emergence of arboviral infections is a challenge to blood transfusion safety. In 2025, the World Health Organisation (WHO) highlighted the need to understand the epidemiology of emerging transfusion‐transmitted infections and their impact on donor selection and blood supply.^1^ It has been estimated that 5.7 billion people live in regions suitable for arbovirus transmission.^2^ These viruses are transmitted through the bite of infected arthropods, mainly mosquitoes, but their potential for transfusion‐transmission has also recently become an important blood safety consideration. The ability of arboviruses to be transmitted via blood transfusion was first demonstrated for West Nile virus (WNV) following its emergence in the United States of America (USA) in 2002^3^ but has since been observed for dengue (DENV) and Zika viruses (ZIKV).^4^ All blood components have the potential to transmit the infection, as demonstrated for WNV,^3^ DENV,^5^, ^6^, ^7^ and ZIKV.^8^

WNV (within the species *Orthoflavivirus nilense*), DENV (*Orthoflavivirus dengue*), ZIKV (*Orthoflavivirus zikaense*), JEV (Japanese encephalitis virus, *Orthoflavivirus japonicum*), USUV (Usutu virus, *Orthoflavivirus usutuense*) all in the family *Flaviviridae* and CHIKV (*Alphavirus chikungunya*; family *Togaviridae*) are all positive‐sense RNA viruses (Table 1). Most infections are asymptomatic, but they can also present with non‐specific symptoms including fever, headache, malaise, rash and fatigue.^9^ Individuals with immune deficiencies, elderly, and pregnant women are more likely to develop severe diseases, including central nervous system involvement with an up to 20% mortality rate.^10^ Approximately 700,000 deaths are estimated to occur annually worldwide due to arboviral infections.^11^

**TABLE 1:** Summary of the transmission modes and epidemiology of the mosquito and tick‐borne infections addressed by the study.

Virus	Family	Vector	Amplification hosts	Endemic areas in both amplification hosts and humans								Spread to non‐endemic regions 2024	Transfusion‐transmitted infections documented
				Caribbean	North America	South America	Asia	Middle East	Pacific Island	Europe	Africa		
Dengue	*Flaviviridae*	*Aedes albopictus* and *Aedes aegypti* mosquitos	Humans in urban cycles and non‐human primates in enzootic cycles	Y	N	Y	Y	N	Y	N	Y	Sporadic cases in France, Italy and Spain	Yes
Zika	*Flaviviridae*	*Ae. albopictus* and *A. aegypti* mosquitos	Humans in urban cycles and non‐human primates in enzootic cycles	Y	N	Y	Y	N	Y	N	Y		Yes
Chikungunya	*Togaviridae*	*Ae. albopictus* and *A. aegypti* mosquitos	Humans in urban cycles and non‐human primates in enzootic cycles	Y	N	Y	Y	N	Y	N	Y	Sporadic case in France	None reported
Japanese Encephalitis	*Flaviviridae*	Aedes and *Culex* species mosquitos	Birds and pigs	N	N	N	Y	N	Y	N	N		Yes
West Nile	*Flaviviridae*	*Culex pipiens* and *Culex modestus* mosquitos	Birds	Y	Y	Y	N	Y	N	Y	Y		Yes
Usutu	*Flaviviridae*	*C. pipiens* and *C. modestus* mosquitos	Birds	N	N	N	N	N	N	Y	Y		No

As arboviral infections can be asymptomatic or mild, it is not possible to defer arbovirus‐infected individuals from blood donation based on their symptoms alone. The measures currently used to minimize the transfusion‐transmission of arboviruses include suspending blood collection in regions where infections occur, deferring donors returning from such regions, implementing donation screening, or applying pathogen reduction. In many jurisdictions, donor deferral based on local emergence of infection or travel history may neither be practical nor possible, especially when the emergence of several arboviral infections occurs simultaneously or in large geographical areas. Although nucleic acid amplification testing (NAT) for blood donation screening is available for WNV RNA on fully automated platforms, novel multiplexed NAT assays for the simultaneous detection of WNV, DENV, ZIKV, and/or CHIKV in blood donations have been developed.^12^, ^13^ The alternative strategy to reduce risk through pathogen reduction can be applied for platelets and plasma, and has been demonstrated to be effective at reducing the infectivity of a number of arboviruses,^14^, ^15^, ^16^ but licensed methods are not currently available for red cell components.^17^

To our knowledge, there has not been a recent review on how blood establishments worldwide address challenges caused by the current rising incidences of arboviral infections or in their preparedness for future emergence. This study provides an international review of the donor deferral strategies and implementation of donation screening and pathogen reduction by different blood establishments in regard to arboviruses. We have included WNV, DENV, ZIKV, and CHIKV due to the high global incidence and/or availability of testing. JEV and USUV were also considered as the JEV vaccine and USUV, both genetically similar to WNV, are co‐detected by some commercial assays used for WNV screening.^18^ The information acquired will improve awareness and support policy development to strengthen capacity building for safeguarding blood supplies against current and future arboviral threats.

## 2 MATERIALS AND METHODS

### 2.1 Survey development

Several co‐investigators developed the survey through multiple meetings; questions were improved according to feedback received. The survey aimed to summarize current screening practices, deferral strategies, and preparedness against emerging arboviral infections, including JEV, WNV, USUV, DENV, ZIKV, and CHIKV. Data were also collated for tick‐borne encephalitis and Crimean‐Congo hemorrhagic fever viruses but have not been included as the focus of the current paper is on mosquito‐borne infections.

The survey comprised six sections which collected general information on blood services, including their use of pathogen reduction, arbovirus screening, and confirmatory testing practices; documented and reported transfusion‐transmitted cases; deferral strategies of blood donors; and information on mosquito control (Supporting Information S2). Respondents were asked to provide data for 2024 only. Ethical approval was not required for this study as the data gathered did not include blood donor or donation identifiers.

### 2.2 Survey distribution

The survey was circulated to members of the International Society of Blood Transfusion (ISBT) Working Party on Transfusion‐Transmitted Infectious Disease (WP‐TTID) in May 2025, and their voluntary participation was encouraged. A reminder email was sent in August 2025. Survey respondents completed the questionnaire in English through Jisc Online Surveys or a Microsoft Word document. If further clarification was required pertaining to a response, the respondents were contacted via email prior to the interpretation of the data gathered.

### 2.3 Data analysis

Descriptive statistics were performed, including calculation of proportions. The geographical locations of the participating blood establishments that screened for WNV were shown using a map generated using Mapchart.net.

## 3 RESULTS

### 3.1 Demographics

Of the more than 50 blood establishments within the WP‐TTID, approximately 40% responded to the survey. Of the 23 respondents (Australia, Brazil, Canada, China, Colombia, England, Finland, Germany, Iran, Japan, Korea, Netherlands, Poland, Romania, Russia, Saudi Arabia, South Africa, Spain, Sweden, Switzerland, and the United States; Table 2), 13 (57%) represented national blood establishments and 10 (43%) represented regional blood establishments. Most establishments were from Europe (*n* = 10; 42%), followed by Asia (*n* = 6; 25%), North America (*n* = 3; 13%), South America (*n* = 2; 8%), Africa (*n* = 1; 4%), and Oceania (*n* = 1; 4%). These included four upper middle‐income and 17 high‐income countries. Data from responding organizations represented a total population of 1.45 billion people and 29.9 million blood donations collected in 2024, excluding plasma donations collected for manufacturing.

**TABLE 2:** Demographic data for blood establishments (*n* = 23).

Blood establishment	Country	Type of service	Population covered (million)	Blood donations in 2024
Australian Red Cross Lifeblood	Australia	National	27.3	894,877
Hospital Israelita Albert Einstein	Brazil	Regional	3.5	15,600
Canadian Blood Services	Canada	National^a^	41.5	805,525
Hong Kong Red Cross	China	Regional	7.53	201,416
Instituto Nacional de Salud	Colombia	National	52.6	997,115
NHS Blood and Transplant	England	National	57	1,492,302
Finnish Red Cross	Finland	National	5.64	170,365
German Red Cross	Germany	National	83.6	5,102,577
Bavarian Red Cross, Germany	Germany	Regional^b^	13.2	473,282
Isfahan blood transfusion	Iran	Regional	4.87	56,000
Japanese Red Cross Society Blood Service	Japan	National	123.7	5,013,064
Korean Red Cross Blood services	Korea	National	51.8	2,639,162
Sanquin Blood Supply Foundation	Netherlands	National^c^	17.9	380,000
Institute of Hematology and Transfusion Medicine	Poland	National	37.4	1,388,000
National Institute of Blood Transfusion	Romania	National	19.1	486,261
Pirogov National Medical Surgical Center	Russia	National	146	2,200,000
King Abdulaziz University Hospital	Saudi Arabia	Regional	3.7	8300
South African National Blood Service	South Africa	National^d^	62	1,033,015
Centro de Transfusion de Madrid	Spain	Regional	7.13	225,650
Region Skåne	Sweden	Regional	1.43	43,870
Interregional Blood Transfusion SRC	Switzerland	Regional	5.55	154,469
Vitalant	USA_1	Regional^e^	340.1	1,204,257
American Red Cross	USA_2	Regional	340.1	4,873,740

^a^ Except Quebec.

^b^ Data is a subset of the total data from Germany.

^c^ Blood donations excluded plasma donations for plasma‐derived medicinal products including pathogen reduced plasma.

^d^ Except Western Cape.

^e^ Vitalant operates in 18 US states but is not the sole operator in each of those states. Both Vitalant and the American Red Cross collected blood in 46 states and the District of Columbia in 2024; the population covered is a cumulative value of both establishments.

### 3.2 Countermeasures implemented by blood services

#### 3.2.1 Temporary donor deferral strategies

A total of 19 (79%) blood establishments temporarily apply blood donor deferrals based on potential travel‐related arbovirus exposure (Table 3). In general, no blood establishments specified donor deferral for USUV or JEV endemic regions. Donor deferrals were specific, although variable, for WNV, DENV, ZIKV, and CHIKV. Typically, the deferral period for the respective viruses, if implemented, was about 28 days (range: 21–180 days). However, deferral based on diagnosed or suspected infection as opposed to travel may differ and is not presented here.

**TABLE 3:** Deferral strategies, in 2024 only, applied by blood establishments against donors returning from endemic regions and when local transmission occurs. All respective viruses include: West Nile virus (WNV), Usutu virus (USUV, in the *Orthoflavivirus usutuense* species), dengue virus (DENV), Chikungunya virus (CHIKV, in the *Alphavirus chikungunya* spicies within the family *Togaviridae*), Zika virus (ZIKV), and Japanese encephalitis virus (JEV, in the *Orthoflavivirus japonicum* species).

Country	Donor deferral from endemic regions						Donor deferral period from endemic regions	Donor deferral when local transmission occurs
	WNV	USUV	DENV	ZIKV	CHIKV	JEV		
Australia	Y	N	Y	Y	Y	N	28 days	DENV
Brazil	Y	Y	Y	Y	Y	N	30 days	None
Canada	Y	N	Y	Y	Y	N	21 days^a^	None
China (Hong Kong)	Y	N	N	N	N	N	28 days	None
Colombia	Y	N	Y	Y	Y	N	1 month	None
England	Y	N	Y	Y	Y	N	28 days	None
Finland	Y	Y	Y	Y	Y	Y	28 days	None
Germany	Y	N	N	Y	Y	N	28 days	WNV
Iran	Y	Y	Y	Y	Y	Y	Unknown	Unknown
Japan	Y	Y	Y	Y	Y	Y	28 days	None
Korea	Y	Y	Y	Y	Y	Y	28 days	None
Netherlands	Y	N	Y	Y	Y	Y	28 days^b^	None
Poland	Y	N	Y	Y	Y	N	28 days	WNV
Romania	Y	Y	Y	Y	Y	Y	28 days^c^	None
Russia	N	N	N	N	N	N	No response	No response
Saudi Arabia	Y	N	N	N	N	N	120 days	DENV
South Africa	N	N	N	N	N	N	N/A	WNV, DENV, CHIKV, and ZIKV
Spain	Y	Y	Y	Y	Y	Y	28 days	WNV
Sweden	Y	N	Y	Y	Y	N	28 days	None
Switzerland	Y	N	Y	Y	Y	Y	28 days	None
USA_1	N/A						N/A	N/A
USA_2							N/A	N/A

^a^ Except for travelers from the United States and Western Europe.

^b^ General deferral of 28 days for all donations including plasma for plasma‐derived medicinal products (PDMPs) for donors after a visit outside of Europe. WNV donors are only deferred for 28 days for visits outside of Europe. DENV deferral was applied for local transmission of DENV within Europe in 2024.

^c^ Twenty eight days for WNV, 6 months for other viruses. N/A = not applicable, and for United States, as donor deferral in the United States is implemented only for those returning from malaria endemic regions, and not for WNV alone.

#### 3.2.2 Pathogen reduction

There were 12 blood establishments (52%) that performed pathogen reduction on certain blood components; 11 of the 17 high‐income countries performed pathogen reduction whereas only one of four upper middle‐income countries conducted pathogen reduction (Table 4). This varied between blood establishments, with implementation applied to 100% or only a small proportion of platelet units.

**TABLE 4:** Arbovirus circulation within the country/region of each blood establishment's country and their non‐travel‐related screening in 2024.

Country	WNV	USUV	DENV	ZIKV	CHIKV	JEV	Pathogen reduction	Screening	Local deferral
Iran	Y	NK	Y	N	Y	N	Unknown	None	None
Colombia	NK	NK	Y	Y	Y	NK	Some RBC and platelets	None	None
Saudi Arabia	N	N	Y	NK	Y	N	Some plasma and all platelets	None	For DENV
Russia	Y	NK	N	N	N	N	Some plasma and platelet donations	None	NK
Spain	Y	NK	N	N	N	N	All plasma and some platelet donations	WNV	For WNV
China HK	N	N	N	N	N	N	Some plasma and platelet donations	None	None
Korea	N	N	N	N	N	Y	Not conducted	None	None
Australia	Y (i)	N	N (ii)	N	N	Y (iii)	Not conducted	None	For DENV
Finland	N	N	N	N	N	N	Not conducted	None	None
Netherlands^a^	N	Y	N	N	N	N	Some plasma donations^a^	None	None
Germany	Y	Y	N	N	N	N	Some plasma and platelet donations	WNV	For WNV
South Africa	Y	N	N	N	N	N	Not conducted	None	Based on local outbreak^b^
Switzerland	NK	NK	N	N	N	N	All platelets and some plasma donations	None	None
England	N	Y	N	N	N	N	Not conducted	None	None
Japan	N	N	N	N	N	Y	Not conducted	None	None
Canada	Y	NK	N	N	N	N	All platelets and some plasma donations	WNV	For WNV
Poland	Y	N	N	N	N	N	Some plasma and platelet donations	None	None
Sweden	N	N	N	N	N	N	Not conducted	None	None
Romania	Y	N	N	N	N	N	Not conducted	WNV	For WNV
Brazil	N	N	Y	Y	Y	N	Not conducted	None	None
United States	Y	N	Y	Y	Y	N	Some plasma, platelets and cryoprecipitate	WNV	For DENV and ZIKV

Abbreviations: CHIKV, *Alphavirus chikungunya*; family *Togaviridae*; DENV, dengue; JEV, Japanese encephalitis virus, *Orthoflavivirus japonicum*; USUV, Usutu virus, *Orthoflavivirus usutuense*; WNV, West Nile virus; ZIKV, Zika viruses. RBC, red blood cells. NK, not known.

^a^ Plasma donations for fractionation and pathogen reduced plasma account for the majority of our plasma collections; some plasma collections are quarantined and not treated by pathogen reduction techonologies. (i) Kunjin strain endemic in areas of Australia, (ii) episodic in some parts of Australia, (iii) endemic in some areas of Australia.

^b^ An outbreak threatening local blood safety would result in a local deferral accordingly based on regular surveillance in collaboration with the National Institute of Communicable Diseases. Local deferral has been implemented for DENV and ZIKV, previously.

#### 3.2.3 Arboviral screening

Based on our survey, routine WNV screening has been implemented by 9 of the 23 respondents (Table 5), all from high‐income countries. The blood establishments in the United States (*n* = 2) implemented universal screening of blood donations collected in their region throughout the year. Spain, Germany, and Romania (*n* = 3) screened based on local endemicity. Whereas three respondents from the Netherlands, Switzerland, and England have implemented additional screening only for donors returning from travel to any WNV endemic regions, and in the case of the Netherlands, only those traveling in WNV affected areas in Europe were tested. Interestingly, Canada does not consider WNV endemicity in their own country while screening donors who had traveled outside of Canada within 8 weeks from their donation.

**TABLE 5:** West Nile virus (WNV) screening and confirmatory procedures of survey respondents who perform testing on blood donations (*n* = 9). The data is reported for 2024.

Country	WNV NAT since (year)	Screening part of routine testing	Screening of returning travelers	Assay used for WNV screening	Pool size used for screening	Number of blood donations collected in 2024	Number of blood donations screened for WNV in 2024	WNV‐positive donations in 2024^a^	Confirmatory testing used for donations found WNV‐positive in screening
Canada	2003	During WNV season	During WNV season	Cobas® ROCHE	6	805,525	518,270	6 (16)	The same sample and another sample from same donation using a *different assay*
England	2012		During WNV season	Cobas® ROCHE	6	1,492,302	57,543	0 (1)	The same sample using a *different assay* and sequence positives
Germany	2020	Yes	During WNV season	Cobas® ROCHE or GRIFOLS or GFE PoET	ID, 96, 48	5,102,577	2,231,725	37 (146)	The same sample using a *different assay* and sequence positives
Netherlands	2020		During WNV season^b^	Cobas® ROCHE	ID	380,000	13,176	0 (3)^c^	The same sample using a *different assay* and sequence positives
Romania	2013	During WNV season	During WNV season	Cobas® ROCHE	ID	486,261	34,276	6	Another sample from the same donation tested with the same assay
Spain	2019	Yes	During WNV season	Cobas® ROCHE	ID	225,650,00	5908	0	Another sample from the same donation tested with the same assay
Switzerland	2018		During WNV season	Cobas® ROCHE	ID	154,469	2321	0	The same sample using a *different assay*
USA_1	2003	Yes	Throughout the year	Grifols	ID, 6, 16	1,204,257	1,203,039	46 (56)	Another sample from the same donation tested with the same assay
USA_2	2003	Yes	Throughout the year	Grifols^d^	ID, 6, 16	4,873,740	4,722,872	161 (167)	Another sample from the same donation tested with the same assay

Abbreviation: NAT, nucleic acid amplification testing.

^a^ WNV‐reactive sample numbers are shown in brackets.

^b^ Test all travelers returning in the previous 28 days from affected areas within Europe.

^c^ Three confirmed as Usutu virus.

^d^ Supplemental testing is conducted with Cobas® ROCHE.

Two blood establishments from the United States indicated that should an arbovirus outbreak occur, they would have a contingency plan to implement NAT, already approved by their Food and Drug Administration (FDA), as seen in the past for DENV or ZIKV. Switzerland's respondent also had preparedness plans for DENV, ZIKV, and CHIKV aiming to implement screening if local transmission were to occur in their region. Furthermore, Poland has developed guidelines for action in the event of a domestic WNV case, including donor deferral and blood donation screening with verified NAT‐based assay.^19^

#### 3.2.4 WNV screening methods and confirmatory testing

**FIGURE 1:**
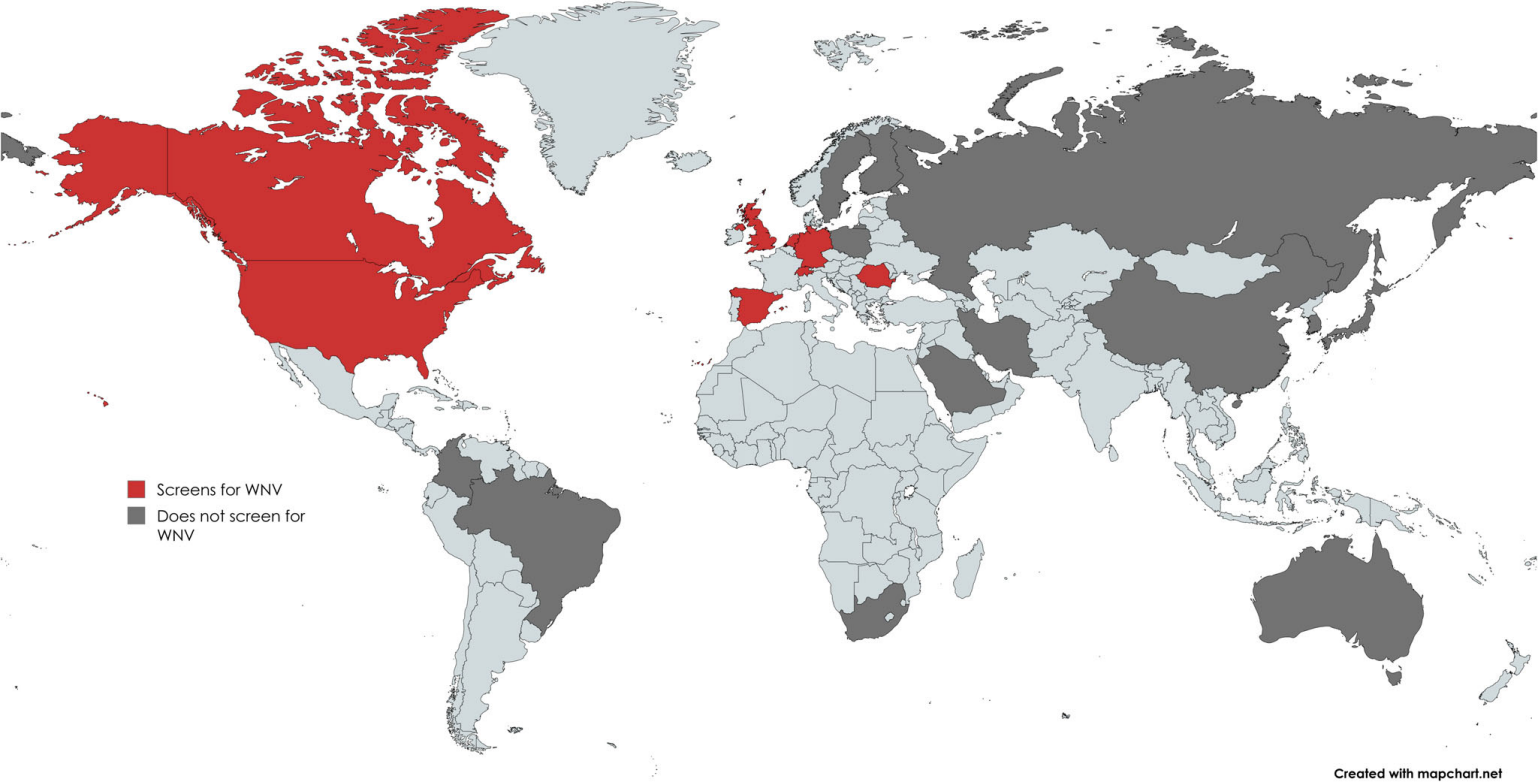
The distribution of each establishment's West Nile virus (WNV) screening practices. Red shading indicates the establishment screens for WNV and gray shading indicates the establishment does not screen for WNV.

WNV screening was performed by nine blood establishments using commercial assays (Table 5, Figure 1). Among those that perform screening, four always tested samples individually (ID‐NAT), two screened samples in pools of six, and the remaining three blood establishments used larger pool sizes. Two blood establishments in the United States stated ID‐NAT was used during the WNV season, whereas samples were screened in pools of six or 16 outside the season. Similarly, the blood establishment from Canada specified that a positive WNV NAT would trigger a change from pooled to individual sample testing of all donations collected within a 100 km radius of the implicated donor for a period of 7 days after detection.

Confirmatory testing was performed by Spain, the Netherlands, Germany, England, Canada, Romania, and the two establishments in the United States. Confirmatory testing included sequencing of amplicons from positive samples providing positive virus identification by blood establishments from the Netherlands, in both German blood establishments^20^ and in England. Switzerland has planned to conduct confirmatory testing when a reactive donation is found.

#### 3.2.5 Arboviral infections among blood donors

Six blood establishments reported initially WNV‐reactive blood donations in 2024: three from the Netherlands, one from England, 146 from Germany, six from Romania, 16 from Canada, and 223 combined from the two blood establishments in the United States (Table 5). All initial WNV‐reactive donations in the Netherlands were subsequently found to be USUV not WNV, whereas 6 of 16 initial WNV reactives from Canada were confirmed as WNV. In the United States, 207 of the 223 WNV screen reactive cases were confirmed as WNV (46 of the 56 for USA_1 and 161 of the 167 for USA_2). Germany had confirmed 37 of their 146 initial WNV‐reactive samples; 31 as WNV with next generation sequencing (NGS) and an additional 6 cases that were confirmed with a second, more specific NAT. Of the non‐confirmed reactives, 36 were found to be USUV and one proved to be JEV vaccine virus by sequencing of the amplicon, while 72 remained indeterminate.^20^ Romania confirmed the six reported cases as WNV‐positives. No confirmed positives were identified by Spain, the Netherlands, Switzerland, and England, despite screening 5908, 13,176, 2321 and 57,543 donations, respectively, from returning travelers in 2024.

Circulation of WNV was evident in four of six countries that reported WNV‐infected blood donors, and we further calculated the WNV incidence per 100,000 blood donations screened in those countries. During 2024, a total of 256 confirmed WNV‐positives were identified by screening of 8,710,209 blood donations (2.9 per 100,000 screened). The incidence of WNV was highest in Romania (6 from 34,276, 17.5 per 100,000), followed by the United States (207 from 5,925,911, 3.5 per 100,000), Germany (37 from 2,231,725, 1.7 per 100,000 screened), and Canada (6 from 518,270, 1.2 per 100,000).

There were no documented or confirmed transfusion‐transmitted arboviral infections reported by the participating blood establishments in 2024.

#### 3.2.6 Future concerns for blood services

Participants were asked to list any other emerging infections they considered to be a potential risk for blood safety that were not addressed by the survey (Supporting Information S1). Of note, one probable case of transfusion‐transmitted Ross River virus infection was reported by Australia in 2014.^21^ Similarly, Poland noted concerns over tick‐borne encephalitis virus (TBEV), following transmission via organ transplantation.^22^

## 4 DISCUSSION

The global epidemiology of mosquito‐borne arboviruses is rapidly changing. There has been a geographical expansion of mosquitos, such as the invasive *Aedes albopictus* into new regions, facilitated by climate change and globalization and the associated spread of viruses vectored by the species.^23^ For example, the expansion of DENV infections was noted in 2024 with 14.1 million cases reported by over 100 countries globally.^24^ In addition, arboviruses have adapted to undergo direct human amplification through creating higher WNV loads in infected humans and enhanced transmission efficiency in mosquitoes.^25^ Hence, they continue to pose challenges for blood safety. To understand how blood establishments deal with emerging arboviral infections, we collected data from various countries involving all continents. A population of 1.45 billion people and over 29 million blood donors in 2024 were covered. Our data shows substantial variability in the main three strategies currently employed to prevent transfusion‐associated arbovirus transmission: donor deferrals based on travel history or local epidemiology, pathogen reduction, and donation screening.

The survey responses show frequent reliance on donor deferrals or testing based on travel history to arboviral endemic regions to exclude viremic donors. Travel‐related donor deferral was implemented by 19 of the 23 respondents, and usually for 28 days. At a time of markedly increasing incidences of arboviral infections, a policy to defer donors based on travel history alone may not be a sustainable strategy for all blood operators and could lead to significant donor loss and blood product shortage. As an example, increased WNV transmission within the United States in 2012 led to the introduction of travel‐related WNV screening of blood donations in the UK to avoid significant frequencies of donor deferrals from returning travelers.^26^ The circulation of multiple arboviruses simultaneously, such as DENV, ZIKV, CHIKV^2^ and Oropouche in Latin America,^27^ adds to the complexity of travel‐related deferrals of blood donors as a deferral for an arbovirus may take precedence even if WNV testing is in place. Blood establishments will need to estimate how the spread of multiple arboviruses into non‐endemic regions affects their donor deferral rates, noting the presence of arboviruses in the travel destination is not the only factor to be considered. Further risk assessment should also consider the type of travel, including where the donors go and what activities they do engage in at the destination. This information will guide how deferral strategies are implemented and whether other methods, such as pathogen reduction or implementation of further donation screening, will also be needed. When additional mitigation strategies are implemented, cost‐effectiveness should also be considered.^8^, ^28^, ^29^

There is also a necessity to consider whether the importation of individual cases will likely lead to local transmission of infection. For example, there was a large epidemic of CHIKV in La Réunion and Mayotte in 2024, 18 years after the first significant outbreak on the islands, with a sudden increase in imported cases to mainland France during this period.^30^ Unfortunately, these cases established local transmission of CHIKV in France where *Ae. albopictus* was already established. Local transmission of arboviruses, in addition to WNV, is predicted to increase in non‐endemic regions such as Northern Europe where the vectors are becoming increasingly established, suggesting further blood donor transmission prevention measures will become necessary.^23^ Rapid implementation of deferral of donors is also necessary within a country or region of the blood establishment when local arboviral transmission occurs, noting only 10 respondents from Saudia Arabia, Spain, Australia, the Netherlands, Germany, South Africa, Switzerland, Canada, Romania, and the United States reported they would defer donors during episodes of local transmission of specific arboviruses.

Another challenge for blood establishments is the identification of risk regions to target for implementation of travel or local transmission‐based donor deferrals. There are commonly delays in the identification, diagnosis, and reporting of human cases of arboviruses.^31^ Better identification of risk areas for the trigger of these measures could be mitigated by One Health surveillance. This includes interventions that combine real‐time entomological, veterinary, and human surveillance data and improved collaboration between human and animal health sectors. Such strategies have been shown to be useful, for example, in Italy for the early detection of WNV and USUV circulation.^32^

Survey responses revealed the frequent use of pathogen reduction methods for platelets and/or plasma, more commonly within high‐income countries in comparison to upper middle‐income countries (65% vs. 25%). Most pathogen reduction technologies have been shown to be effective against enveloped viruses, including arboviruses, but their applicability is currently largely limited to platelets and plasma. Pathogen reduction might be a useful strategy, especially in lower and middle‐income countries with greater arbovirus endemicity, subject to cost and provided these limitations can be overcome.

Few countries screened for arboviruses directly, and where conducted, only for WNV. WNV screening used a variety of commercial assays for viral RNA detection.^33^ These were variably conducted on individual samples, pools of 6, 16, 48, or 96, depending on the assay used. Screening of blood donations for WNV was first introduced by the United States and Canada in 2003, as a result of the documented transfusion‐transmitted WNV infections in blood recipients.^34^ Since then, the large‐scale WNV outbreaks in neighboring regions or the identification of the locally acquired WNV cases have prompted other blood establishments to implement either universal or travel‐related WNV screening. In Romania, the first WNV cases were reported in 1996 and the subsequent re‐emergence of WNV with a culminating large outbreak in 2010 led to the implementation of blood donation screening in 2013.^35^ Although WNV has predominantly affected Southern Europe, it has recently started to spread northwards. The first locally acquired human cases in Northern Europe were reported in Germany in 2019^36^ and the Netherlands in the following year, leading to the implementation of donation screening in both countries in 2020. Furthermore, WNV screening of blood donors has not been implemented outside of North America or Europe despite its much more extensive circulation in several countries in Africa, including Uganda, Algeria, Central African Republic, Egypt, Côte d'Ivoire, Kenya, Morocco, Tunisia, Senegal, South Africa, Botswana, Congo, Djibouti, Madagascar, Mozambique, Namibia, and Tanzania.^37^

Confirmation of reactivity in WNV RNA‐screening assays is necessary and widely performed. Many commercial assays are known to detect all members of the JEV serocomplex, and hence USUV infections and JEV may be falsely reported as WNV without further discriminatory tests. This is highlighted by data from the Netherlands and Germany; in the Netherlands, all initial WNV‐reactive positive donations were shown to be USUV. In Germany, 36 initially WNV‐reactive donations were shown to be USUV and one was identified as the inactivated JEV vaccine strain. This is comparable to JEV vaccine strain detection in several donors screened in Canada in 2019^38^ and one case in the UK in 2024 (unpublished data).

WNV screening implemented in four endemic countries, the United States, Canada, Romania, and Germany, successfully identified and intercepted 256 RNA‐positive blood donations in 2024. Although blood donation screening of WNV was not implemented inclusively in all endemic countries, no transfusion‐transmitted WNV infections were reported in 2024. Similarly, no transfusion‐transmitted cases of DENV were reported in this study despite its high endemicity in Iran, Colombia, Saudi Arabia, and Brazil.

Depending on the local epidemiology, the lack of transfusion‐transmitted arboviral infections reported in this study could reflect the possible inability of some arboviruses such as CHIKV to transmit via blood transfusion, despite the viremic blood donors.^4^ High frequencies of past exposure to CHIKV or other antigenically cross‐reactive alphaviruses in blood recipients may protect from transfusion‐transmitted infections. Although 2.1% of blood donors were found to be positive for CHIKV RNA during a 2014 chikungunya epidemic in Puerto Rico, United States,^39^ and similarly, 14.5% tested positive for DENV RNA during the Dengue epidemic in Columbia in 2019, no transfusion‐transmitted infections were reported by either of those studies.^40^ However, it is important to note that while there is limited data on how well arboviruses remain infectious in stored blood components, clinicians may not consider blood transfusion as a possible source of any arbovirus infection, especially in higher prevalence settings or during an outbreak.

A limitation of the study was the lack of survey responses from low‐ to middle‐income countries. While the overall response rate was high, it was biased toward more high‐income countries and could affect generalization of the results. However, the proportion of respondents performing WNV blood donation screening was similar to a previous NAT survey,^33^ where all WNV screening reported to be conducted was also by blood establishments within North America and Europe. Similarly, South American or African regions did not screen for WNV and were also less likely to apply any NAT for blood donation screening. The best practice for a blood establishment to follow for arboviral infections will depend on the local epidemiology, donor travel patterns, and the specific arboviruses. The factors can be inferred through risk assessments, and this has not been addressed by the survey.

In conclusion, blood safety protective measures currently implemented against arboviral infections are mostly limited to donor deferrals based on travel history. Despite the spread and potential for arboviruses such as DENV, CHIKV, and ZIKV to transmit through blood transfusion, screening of donors for these pathogens is not currently conducted. As arboviruses have multiple vertebrate and invertebrate reservoir hosts, the control of these viruses can be difficult. While mosquito control efforts such as the use of *Wolbachia* bacteria to combat DENV show great promise for the future,^41^ it is essential for blood services to be prepared for the potential spread of arboviruses. This study underscores the importance of capacity building and preparedness strategies by blood establishments, which will be further supported by technical guidelines being developed for the prevention of emergent arbovirus transmission through substances of human origin by the European Centre for Disease Control. There is a need to strengthen One Health surveillance capabilities, which could further inform the need for mitigation strategies and improve outbreak preparedness.

## CONFLICT OF INTEREST STATEMENT

The author has disclosed no conflicts of interest.

## Supporting information


**Data S1.** Supplementary Information.

**Data S2.** Supplementary Information.

## Data Availability

The data that support the findings of this study are available from the corresponding author upon reasonable request.
