# Supplementary material for: An international study on emerging arboviral infections and blood safety
Source: Transfusion. 2026 Jun 18;66(9):1786–99. doi: 10.1111/trf.70306 (PMC13569194; doi:10.1111/trf.70306)
Supplement: Supplementary file 1 [file TRF-66-1786-s002.pdf]

Emerging arboviral infections and blood safety

Emerging and re-emerging arboviral infections pose a risk for blood safety. This is due to potential direct transmission via the blood supply or through the loss of previously eligible blood donors. More recently there has been an increase in human cases and the spread of these viruses from endemic regions to new areas. Most arboviral infections result in asymptomatic viraemia, but if transfused, transmitted infections can create severe clinical implications in the immunocompromised and the elderly.

Mosquito borne viruses spread quickly and have been responsible for outbreaks in various regions of the globe. Examples include Japanese encephalitis virus (JEV), West Nile virus (WNV), Usutu virus (USUV), Dengue virus (DENV), Zika virus (ZIKV) and Chikungunya virus (CHIKV). Human cases caused by tick-borne viruses such as tick-borne encephalitis virus (TBEV) and Crimean-Congo haemorrhagic fever virus (CCHFV) have been more frequently detected, in including in non-endemic regions.

This 10-minute questionnaire is directed at blood establishments worldwide. The survey aims to evaluate how blood services prepare for emerging infections, how they currently tackle issues involved in implementation of testing (*eg.* for WNV and other arboviruses) and policies for donor deferral), the assays used for arbovirus testing (screening and confirmatory testing) and any available information on the incidence and seroprevalence of these infections in their country/region

In this survey, please supply complete data for the calendar year 2024.

All information collected will be kept confidential, and data will be presented without identifying information. You will be included as a co-author in any publications using this data.

We aim to publish these findings, which will strengthen the ability of blood services to respond better to these emerging arboviral threats.

Please email Piya Rajendra (lokuwattage.rajendra@seh.ox.ac.uk) and Prof Heli Harvala (heli.harvala@utu.fi) with any queries.

Thank you for taking the time to complete this survey by the 31.05.25.

Demographic information

*Mandatory

Please supply data for the year 2024.

1. ***What is the name of the blood establishment you work with?**

2. ***Please specify if data is supplied from a whole country or region?**

☐Whole country

☐Region

3. ***Please provide the name of country.**

4. ***Please provide the name of region.**

5. ***What was the total number of residents in your country/region in 2024?**

6. ***How many blood donations have been donated at your blood establishment in 2024 (excluding source plasma donors)?**

7. ***Does your establishment conduct pathogen reduction (PR) on blood components? (Please tick all that apply). Examples of PR include: Solvent detergent, Amotosalen + UV-A (Intercept, Cerus), Methylene blue + light and Riboflavin + UV-B (Mirasol).**

	Yes, to all	Yes, to some	Not known	Not conducted
Red blood cells	☐	☐	☐	☐
Plasma	☐	☐	☐	☐
Platelets	☐	☐	☐	☐
Other	☐	☐	☐	☐

8. **Please specify 'other' blood components where pathogen reduction is conducted?**

9. **If pathogen reduction is not conducted, do you plan to implement this? Please specify.**

Nucleic acid testing (NAT) for blood donors

Data for the year 2024.

10. ***Do you conduct NAT on blood donations for any of the following viruses: Japanese Encephalitis virus (JEV), West Nile virus (WNV), Usutu virus (USUV), Dengue virus (DENV), Zika virus (ZIKV), Chikungunya virus (CHIKV), Tick-Borne Encephalitis virus (TBEV) and Crimean-Congo haemorrhagic fever virus (CCHFV)?**

☐Yes

☐No

11. **Indicate which viruses are screened in blood donor samples using NAT. Please tick all that apply in the table on page 5.**

	No	Yes, throughout the year	Yes, seasonally	Yes, for all donors	Yes, for donors who have returned from endemic regions	Other
West Nile virus	☐	☐	☐	☐	☐	☐
Usutu virus	☐	☐	☐	☐	☐	☐
Japanese Encephalitis virus	☐	☐	☐	☐	☐	☐
Dengue virus	☐	☐	☐	☐	☐	☐
Zika virus	☐	☐	☐	☐	☐	☐
Chikungunya virus	☐	☐	☐	☐	☐	☐
Tick-Borne Encephalitis virus	☐	☐	☐	☐	☐	☐
Crimean-Congo haemorrhagic fever virus	☐	☐	☐	☐	☐	☐

12. **If applicable, please specify any other viruses screened.**

13. **If NAT is conducted on blood donations, please state year of introduction for each respective virus.**

14. **Methods used for NAT screening of blood donations. Please tick all that apply.**

	Not applicable	Commercial assay	In-house assay	Pooled samples	Individual samples
West Nile virus	☐	☐	☐	☐	☐
Usutu virus	☐	☐	☐	☐	☐
Japanese Encephalitis virus	☐	☐	☐	☐	☐
Dengue virus	☐	☐	☐	☐	☐
Zika virus	☐	☐	☐	☐	☐
Chikungunya virus	☐	☐	☐	☐	☐
Tick-Borne Encephalitis virus	☐	☐	☐	☐	☐
Crimean-Congo haemorrhagic fever virus	☐	☐	☐	☐	☐

15. **Please identify the manufacturers providing commercial NAT assays for each virus screened.**

16. **Please provide details and/or a published reference for in-house NAT assays for each screened virus.**

17. **Please provide the number of samples per pool for each screened virus. If individual sample NAT is used, indicate as N=1.**

18. **If applicable, what triggers the conversion of pooled sample NAT to individual sample NAT? Please explain.**

Arbovirus detection and transmissions via blood transfusion

Data on the prevalence of JEV, WNV, USUV, DENV, ZIKV, CHIKV, TBEV and CCHFV in your region/country in 2024.

19. **What is the total number of donations screened for arboviruses in <u>2024</u>, please provide numbers for each virus screened.**

20. **How many donors tested positive for the respective viruses in <u>2024?</u>**

21. **Have there <u>ever</u> been confirmed or documented cases of transfusion transmitted arbovirus infections at your region or country, for each of the respective viruses below? Please select all that apply (table on page 10).**

	Confirmed transmissions via blood transfusion	Unconfirmed transmissions via blood transfusion	No documented transmissions via blood transfusion	Not applicable
West Nile virus	☐	☐	☐	☐
Usutu virus	☐	☐	☐	☐
Japanese Encephalitis virus	☐	☐	☐	☐
Dengue virus	☐	☐	☐	☐
Zika virus	☐	☐	☐	☐
Chikungunya virus	☐	☐	☐	☐
Tick-Borne Encephalitis virus	☐	☐	☐	☐
Crimean-Congo haemorrhagic fever virus	☐	☐	☐	☐

22. **Please state the number of documented and confirmed cases of transfusion transmitted arbovirus infections at your region or country, for each respective virus above.**

Confirmatory testing for positive blood donors

23. **Do you conduct confirmatory testing on NAT positive blood donor samples?**

☐Yes

☐No

Please select **all** that apply for confirmatory testing (table on page 12).

	Not applicable- do not screen	No confirmatory testing	Test the same sample using a different NAT assay	Test a different sample from the same donation with the same NAT	Test a different sample from the same donation with a different NAT	Test a follow-up sample
West Nile virus	☐	☐	☐	☐	☐	☐
Usutu virus	☐	☐	☐	☐	☐	☐
Japanese Encephalitis virus	☐	☐	☐	☐	☐	☐
Dengue virus	☐	☐	☐	☐	☐	☐
Zika virus	☐	☐	☐	☐	☐	☐
Chikungunya virus	☐	☐	☐	☐	☐	☐
Tick-Borne Encephalitis virus	☐	☐	☐	☐	☐	☐
Crimean-Congo haemorrhagic fever virus	☐	☐	☐	☐	☐	☐

24. **Do you conduct sequencing on positive samples?**

	Yes	No	Not applicable
West Nile virus	☐	☐	☐
Usutu virus	☐	☐	☐
Japanese Encephalitis virus	☐	☐	☐
Dengue virus	☐	☐	☐
Zika virus	☐	☐	☐
Chikungunya virus	☐	☐	☐
Tick-Borne Encephalitis virus	☐	☐	☐
Crimean-Congo haemorrhagic fever virus	☐	☐	☐

Deferral strategies for blood donors against arboviral infections

25. **Please select all options that apply to your blood establishment.**

	Donor deferral for donors returning from endemic regions	NAT screening for donors returning from endemic regions	Local human transmission has been reported	Donor deferral when local transmission occurs	NAT screening for donors when local transmission occurs
West Nile virus	☐	☐	☐	☐	☐
Usutu virus	☐	☐	☐	☐	☐
Japanese Encephalitis virus	☐	☐	☐	☐	☐
Dengue virus	☐	☐	☐	☐	☐
Zika virus	☐	☐	☐	☐	☐
Chikungunya virus	☐	☐	☐	☐	☐
Tick-Borne Encephalitis virus	☐	☐	☐	☐	☐
Crimean-Congo haemorrhagic fever virus	☐	☐	☐	☐	☐

26. **If you do defer donors returning from endemic countries, for how long? Please specify for each of the respective viruses above.**

One health surveillance

27. **What is the name of your national public health agency?**

28. **Is vector and/or animal host surveillance conducted in your country or region? (E.g. in mosquitos, ticks, birds, horses and humans).**

☐Yes

☐No

29. **Virus detection in vectors (mosquitos or ticks), sentinel animals and humans.** Please select **all** options that apply (Table on page 16).

	Virus has been detected in vectors	Virus has not been detected in vectors	Not known if virus has been detected in vectors	Virus has been detected in animals	Virus has not been detected in animals	Not known if virus has been detected in animals	Virus has been detected in humans	Virus has not been detected in humans	Not known if virus has been detected in humans
West Nile virus	☐	☐	☐	☐	☐	☐	☐	☐	☐
Usutu virus	☐	☐	☐	☐	☐	☐	☐	☐	☐
Japanese Encephalitis virus	☐	☐	☐	☐	☐	☐	☐	☐	☐
Dengue virus	☐	☐	☐	☐	☐	☐	☐	☐	☐
Zika virus	☐	☐	☐	☐	☐	☐	☐	☐	☐
Chikungunya virus	☐	☐	☐	☐	☐	☐	☐	☐	☐
Tick-Borne Encephalitis virus	☐	☐	☐	☐	☐	☐	☐	☐	☐
Crimean-Congo haemorrhagic fever virus	☐	☐	☐	☐	☐	☐	☐	☐	☐

30. **Is mosquito control implemented in your region/country?** Examples of vector control include adulticides, larvicides and insect growth regulators.

☐Yes

☐No

☐Not known

31. **Please specify the type of vector control used if implemented.**

32. **Are there other emerging infections, not mentioned in this survey, considered a risk to blood safety by your establishment?**

End of survey

33. **Please provide your name and email address.**

34. **Are you happy to be included as a co-author in a publication and if necessary to receive further questions based on your answers?**

☐Yes

☐No

35. **Do you have any comments regarding the survey?**

Thank you for completing this survey, please email a completed copy to lokuwattage.rajendra@seh.ox.ac.uk . If you have any queries please email lokuwattage.rajendra@seh.ox.ac.uk and heli.harvala@utu.fi.
