# Supplementary material for: An international study on emerging arboviral infections and blood safety
Source: Transfusion. 2026 Jun 18;66(9):1786–99. doi: 10.1111/trf.70306 (PMC13569194; doi:10.1111/trf.70306)
Supplement: Supplementary file 2 [file TRF-66-1786-s001.docx]

**Supplementary data**

Information on mosquito control

From the 23 blood establishments, 18 stated that mosquito control had been implemented in their regions/countries whilst 5 blood establishments were uncertain if was used. The most common form of mosquito control was the use of chemical control such as larvicides.

Future concerns of blood establishments

The respondent from Australia indicated other arboviruses, Ross River virus (RRV), Murray Valley encephalitis virus and Barmah Forest virus, have been detected in humans however they represented a low risk to blood safety in Australia, with no reported cases of transfusion transmission apart from one probable case of RRV in 2014 (1). Respondents from Canada and the USA indicated concerns regarding Eastern equine encephalitis virus and the California serogroup viruses. The respondents from the USA stated also concerns about Jamestown Canyon virus, La Crosse encephalitis virus, St. Louis encephalitis virus, Oropouche virus and live Yellow Fever vaccine virus. Powassan and Colorado tick fever viruses were highlighted as a concern by the USA respondents. Poland’s respondent stated haemorrhagic fever viruses, TBEV(2), HEV and *Babesia mictoti* could be a concern. Sweden’s respondent indicated the zoonotic hepatitis viruses, such as human hepatitis E virus, are considered a risk. Furthermore, parasites such as *Babesia* were mentioned by three respondents: China (Hong Kong), the Netherlands and England. In addition, the *Plasmodium* species were noted by respondents from Poland and Colombia. A bacterial infection, *Rickettsia* *africae conorri*, was noted by South Africa’s respondent.

1.	Hoad VC, Speers DJ, Keller AJ, Dowse GK, Seed CR, Lindsay MD, et al. First reported case of transfusion‐transmitted Ross River virus infection. Medical Journal of Australia. 2015;202(5):267–9.

2.	Lipowski D, Popiel M, Perlejewski K, Nakamura S, Bukowska-Ośko I, Rzadkiewicz E, et al. A cluster of fatal tick-borne encephalitis virus infection in organ transplant setting. The Journal of infectious diseases. 2017;215(6):896–901.
